# Real-World Evidence as a Driver of Precision Oncology and Personalized Therapeutic Sequencing in Later-Line Metastatic Colorectal Cancer

**DOI:** 10.3390/cancers18193174

**Published:** 2026-10-01

**Authors:** Carlo Signorelli

**Affiliations:** Medical Oncology Unit, Santa Rosa Hospital, Azienda Sanitaria Locale Viterbo, 01100 Viterbo, Italy; carlo.signorelli@asl.vt.it

**Keywords:** metastatic colorectal cancer, real-world evidence, precision oncology, therapeutic sequencing, biomarker-driven therapy, molecular profiling, circulating tumor DNA, comparative effectiveness research

## Abstract

**Background:** The therapeutic landscape of refractory metastatic colorectal cancer (mCRC) has expanded with antiangiogenic agents, cytotoxic therapies, immunotherapy, and biomarker-driven treatments. Randomized clinical trials (RCTs) establish efficacy and safety, while real-world evidence (RWE) can complement them by addressing generalizability, treatment delivery, safety, and outcomes in populations underrepresented in prospective studies. This narrative review examines the contribution and limitations of RWE in precision oncology and later-line therapeutic sequencing in mCRC. **Methods:** We reviewed evidence from randomized trials, observational studies, real-world registries, translational research, and contemporary international guidance, focusing on later-line antiangiogenic strategies, trifluridine/tipiracil-based treatment, fruquintinib, anti-EGFR rechallenge, immunotherapy in MSI-H/dMMR disease, and targeted therapies for actionable molecular alterations. Particular attention was given to RWE methodology and interpretation. **Results:** RWE complements randomized evidence by characterizing effectiveness and toxicity in heterogeneous populations, treatment delivery and dose modification, and implementation of biomarker-guided strategies in routine practice. Observational studies may also inform therapeutic sequencing and identify prognostic or pharmacodynamic correlates, although causal interpretation is limited by selection bias, treatment attrition, time-dependent and residual confounding. Longitudinal circulating tumor DNA (ctDNA) assessment is particularly relevant to molecular reassessment and anti-EGFR rechallenge, while broader ctDNA-guided adaptive treatment strategies remain investigational. **Conclusions:** Later-line mCRC management increasingly integrates molecular profiling, treatment history, patient fitness, toxicity, and therapeutic sequencing. High-quality RWE provides complementary evidence on how these strategies perform and are implemented in routine practice but should be interpreted according to study design and susceptibility to bias. Integration of rigorous RWE with randomized and prospective molecular evidence may help bridge the gap between trial efficacy and real-world effectiveness and support individualized later-line care.

## Simple Summary

Patients with metastatic colorectal cancer who have exhausted standard treatments now have several later-line treatment options, making treatment selection and sequencing increasingly complex. Randomized clinical trials remain the foundation for these decisions, but trial populations do not always reflect patients seen in routine practice, who may be older, frailer, heavily pretreated, or affected by relevant comorbidities. Real-world evidence can complement trial data by showing how treatments are delivered in everyday care, how toxicity and dose modifications influence treatment exposure, and whether patients remain fit for subsequent therapies. It can also inform the implementation of molecular profiling and circulating tumor DNA testing. However, real-world studies have important methodological limitations and should complement, rather than replace, randomized evidence. This review discusses how these different sources of evidence can support more individualized later-line treatment strategies.

## 1. Introduction

Colorectal cancer (CRC) remains a leading cause of cancer-related morbidity and mortality worldwide. According to GLOBOCAN 2022 estimates, CRC accounted for approximately 1.93 million new cases and more than 900,000 deaths globally [1].

Over the past two decades, metastatic CRC (mCRC) treatment has evolved through combination chemotherapy, antiangiogenic and anti-EGFR agents, immune checkpoint inhibitors, and biomarker-driven targeted therapies. Routine assessment of *RAS*, *BRAF*, HER2 and MSI/MMR status, supplemented when appropriate by next-generation sequencing (NGS), has progressively integrated molecular profiling into treatment selection [2,3,4]. Circulating tumor DNA (ctDNA) can further complement tissue profiling by enabling minimally invasive reassessment of tumor evolution and acquired resistance, although its clinical utility varies according to setting and application [5,6].

Later-line treatment has consequently become an integral component of the therapeutic continuum. Regorafenib, trifluridine/tipiracil, fruquintinib and biomarker-driven strategies—including immunotherapy, HER2- and *BRAF*-directed therapies, *KRAS G12C* inhibition, and ctDNA-informed anti-EGFR rechallenge—have expanded therapeutic opportunities after failure of standard treatment [7,8,9,10,11,12,13,14]. Their availability has shifted the clinical challenge from selecting an individual drug toward integrating treatments across the patient trajectory according to molecular profile, previous exposure, residual fitness, cumulative toxicity, disease characteristics, and patient goals.

Randomized controlled trials (RCTs) remain the standard for establishing efficacy and safety but cannot fully address the heterogeneity of routine practice or all clinically relevant sequencing questions. Older or frail patients and those with comorbidities or complex treatment histories are frequently underrepresented, while dose optimization, treatment attrition, cumulative toxicity, and sequential treatment delivery may be incompletely characterized [15,16]. Real-world evidence (RWE) can complement randomized evidence in these areas, but observational findings require cautious interpretation because treatment selection, access to molecular testing, attrition, and residual confounding may influence observed outcomes [17,18,19,20,21].

**Figure 1:**
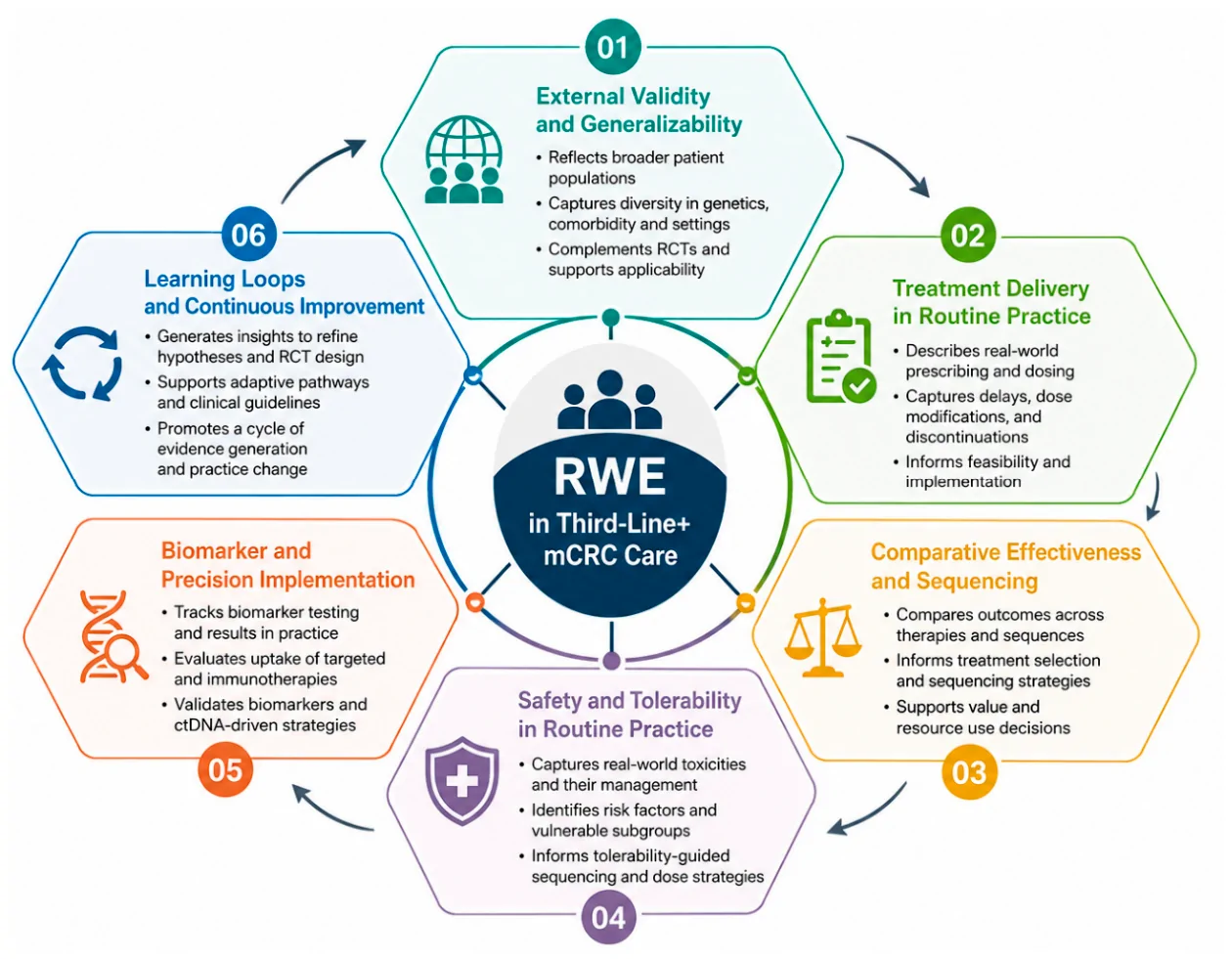
Real-world evidence as a driver of precision oncology in third-line+ metastatic colorectal cancer: six operational domains. Schematic overview of the six domains through which real-world evidence (RWE) informs third-line+ metastatic colorectal cancer (mCRC) care: external validity, treatment delivery, comparative effectiveness/sequencing, safety in routine practice, biomarker implementation and learning loops. The framework is aligned with ESMO-GROW concepts to support interpretability across chapters. Abbreviations: ctDNA, circulating tumor DNA; mCRC, metastatic colorectal cancer; RCT, randomized controlled trial; RWE, real-world evidence.

In this review, the contribution of RWE to third-line and later (3L+) mCRC is considered across six domains: external validity, treatment delivery, comparative effectiveness and sequencing, safety in routine practice, biomarker implementation, and learning loops (Figure 1). These domains provide a framework for distinguishing settings in which RWE can directly characterize routine-care effectiveness and implementation from those in which observational associations remain hypothesis-generating and require prospective validation.

RWE reporting and interpretation are considered within the ESMO-GROW framework and contextualized within contemporary mCRC continuum-of-care recommendations [2,22].

This narrative review integrates pivotal randomized trials, prospective and retrospective observational studies, registries, translational research, and international clinical practice guidelines identified through PubMed/MEDLINE, reference screening, and guideline review. The literature search included publications available up to August 2026 and focused on later-line treatment, therapeutic sequencing, treatment delivery, toxicity, molecular profiling, and biomarker-guided strategies. No formal systematic-review or meta-analytic methodology was applied.

**Figure 2:**
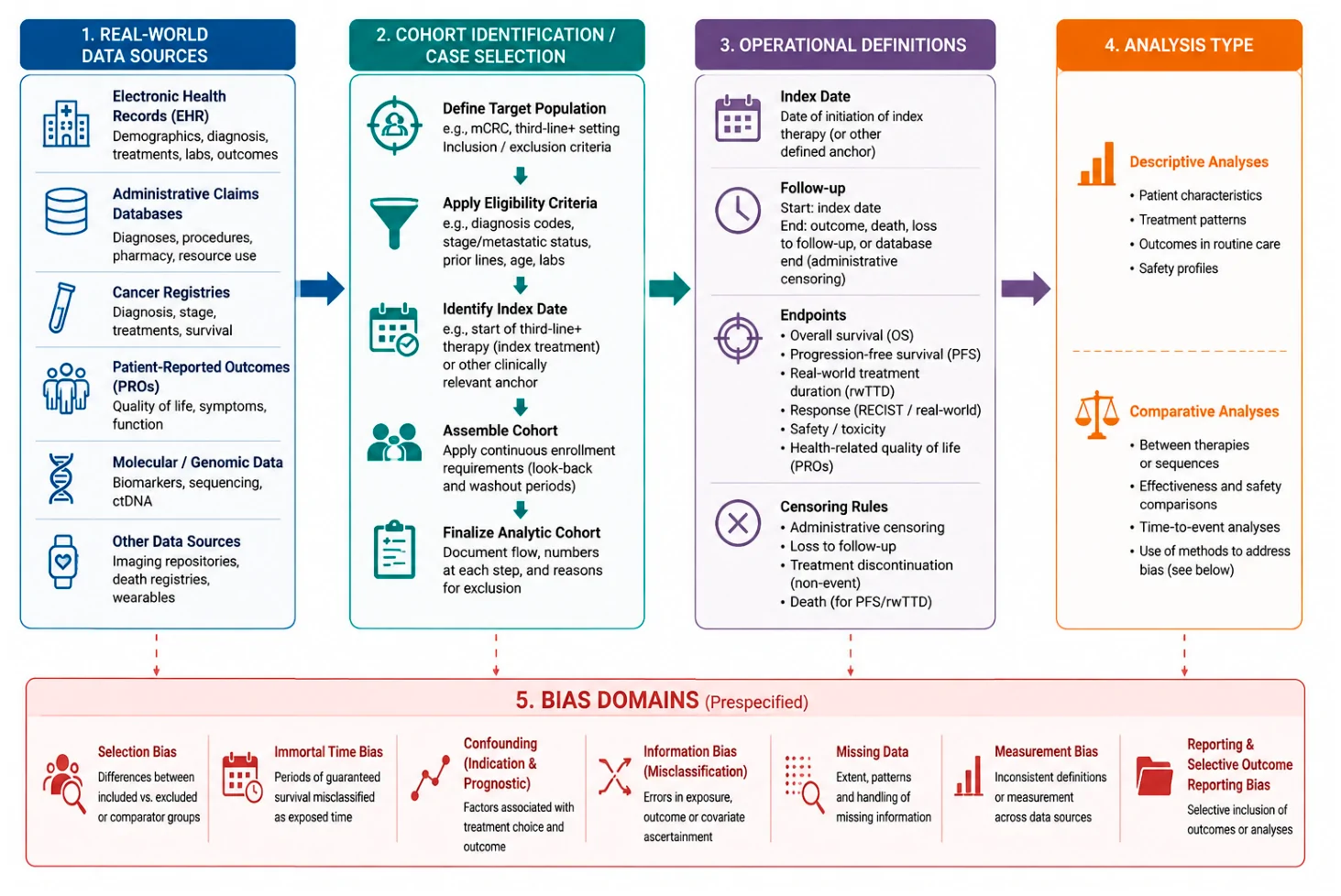
ESMO-GROW-aligned flowchart for oncology real-world evidence: data sources, case selection and key bias domains. Illustrative flowchart linking real-world data sources to cohort identification/case selection, operational definitions (index date, follow-up, endpoints and censoring) and prespecified bias domains (including selection mechanisms and missing data). The structure follows ESMO-GROW flowchart principles and can be adapted to descriptive or comparative analyses. Abbreviations: ctDNA, circulating tumor DNA; EHR, electronic health record; mCRC, metastatic colorectal cancer; OS, overall survival; PFS, progression-free survival; PROs, patient-reported outcomes; RECIST, Response Evaluation Criteria in Solid Tumors; rwTTD, real-world time to treatment discontinuation.

RWE studies were critically interpreted according to transparency of data sources and endpoint definitions, handling of missing data, selection mechanisms, attrition, and reported bias-mitigation strategies. Methodologically robust studies were given greater interpretative weight, whereas observational series with limited control of bias were considered primarily descriptive or hypothesis-generating rather than causal comparative evidence [22]. The corresponding ESMO-GROW-aligned methodological framework is summarized in Figure 2.

Against this methodological background, we examine where RWE can meaningfully complement randomized evidence in later-line mCRC, where its conclusions remain uncertain, and how clinical, molecular, and treatment-delivery information may support individualized therapeutic strategies.

## 2. Why Real-World Evidence Matters

Randomized controlled trials (RCTs) remain the reference standard for establishing the efficacy and safety of anticancer therapies. However, their strict eligibility criteria inevitably select patient populations that may differ from those encountered in routine practice, particularly in later-line metastatic colorectal cancer (mCRC), where older age, comorbidities, impaired performance status, organ dysfunction, and complex treatment histories are common [2,3,15,17].

Real-world evidence (RWE) can complement randomized evidence by addressing questions that are difficult to capture within registration trials. Real-world data may derive from observational cohorts, registries, electronic health records, administrative and claims databases, pharmacy data, patient-reported outcomes, and digital health sources [17,18,23,24,25,26]. In later-line mCRC, these data are particularly useful for describing treatment delivery, dose modifications and discontinuation, safety in less-selected populations, and the feasibility of subsequent therapies [18,24,26,27].

RWE has contributed to the clinical understanding of established later-line treatments such as regorafenib, trifluridine/tipiracil, and fruquintinib, particularly in areas that remain incompletely addressed by pivotal trials. These include treatment sequencing, dose optimization, outcomes in older or frail patients, management of toxicity, and the clinical significance of treatment-emergent markers [7,21,28,29,30,31,32]. Importantly, however, the strength of these observations depends on study design and on the question being addressed. Describing treatment patterns or safety in routine practice requires a different level of inference from comparing therapeutic sequences or attributing improved outcomes to a biomarker-guided strategy.

This distinction is particularly relevant because observational studies are vulnerable to confounding by indication, selection bias, immortal-time and other time-dependent biases, information bias, missing data, and treatment attrition. These limitations cannot always be eliminated by increasing sample size and should be considered when interpreting apparently favorable outcomes in selected populations. This is especially important for patients undergoing extensive molecular profiling or repeated biomarker assessment, who may differ from the broader mCRC population in fitness, access to specialized care, and availability of subsequent treatments [33,34,35].

Methodological approaches such as multivariable adjustment, propensity-score methods, inverse probability weighting, multiple imputation, sensitivity analyses, and, when appropriate, instrumental-variable methods can strengthen observational analyses [33,34,35]. Target-trial emulation provides an additional framework by defining eligibility criteria, treatment strategies, follow-up, endpoints, and the analysis plan of the hypothetical randomized trial before applying these principles to observational data [36,37]. Such approaches can reduce important sources of bias, but they do not make observational evidence equivalent to randomization.

RCTs and RWE should therefore be regarded as complementary rather than competing sources of evidence. Randomized trials establish treatment efficacy under controlled conditions, whereas well-designed real-world studies can clarify how treatments are delivered and perform across more heterogeneous populations. Their contribution to comparative effectiveness, sequencing, and precision oncology is potentially important, but conclusions in these areas should remain proportional to the quality of the underlying data and the ability of the study design to address confounding and selection [17,20,21].

## 3. Evolution of the Therapeutic Landscape Beyond Second-Line Treatment

### 3.1. From Salvage Therapy to a Planned Therapeutic Continuum

Over the past two decades, the management of metastatic colorectal cancer (mCRC) has moved from a line-by-line approach toward a therapeutic continuum, as improvements in earlier treatment have allowed more patients to remain eligible for subsequent therapies. Current guidelines reflect this evolution and place later-line treatment within an overall strategy that also incorporates supportive care [2,3]. Historically, however, treatment options after fluoropyrimidines, oxaliplatin, irinotecan, and biologic therapy were limited [38].

This changed with the development of agents specifically evaluated in refractory disease. Regorafenib improved overall survival in the phase III CORRECT trial [7], with subsequent confirmation in the Asian population enrolled in CONCUR [8]. Trifluridine/tipiracil provided a further active option through RECOURSE [9], while FRESCO-2 more recently established the efficacy of fruquintinib in heavily pretreated patients [10]. Together, these trials established later-line therapy as an evidence-based component of mCRC management rather than an exceptional salvage intervention [1,2].

As more treatments have become available, the clinical question has shifted from whether further therapy should be offered to how available options should be used over time. Direct comparative sequencing data remain limited, and treatment order therefore needs to take into account previous exposure, residual toxicity, patient fitness, disease characteristics, and treatment goals [38,39]. Later-line therapy is consequently better viewed as part of long-term treatment planning, with attention not only to survival but also to preservation of function, quality of life, and the opportunity to receive subsequent therapies [1,2].

### 3.2. Molecular Stratification and the Emergence of Precision Oncology

In parallel with the expansion of later-line options, advances in tumor biology have established mCRC as a molecularly heterogeneous disease. Biomarker assessment is now an integral part of treatment planning throughout the disease course [2,3]. Current guidelines recommend assessment of *KRAS* and *NRAS*, *BRAF V600E*, HER2, and mismatch repair (MMR)/microsatellite instability (MSI) status, while multigene next-generation sequencing (NGS) can identify less common actionable alterations, including *KRAS G12C*, NTRK and *RET* fusions, and *POLE*/*POLD1* proofreading-domain variants [2,3,4].

This molecular stratification has expanded treatment opportunities beyond second-line. Biomarker-directed strategies are now available for selected patients with *BRAF V600E*-mutated, HER2-positive, *KRAS G12C*-mutated, MSI-H/dMMR, and other less common molecularly defined tumors [1,2,4]. The clinical meaning of a biomarker, however, depends on the evidence supporting its use. Predictive biomarkers identify differential likelihood of benefit from a particular treatment, whereas prognostic biomarkers provide information on outcome independently of treatment. Pharmacodynamic markers reflect biological effects of drug exposure and should not be regarded as predictive without appropriate validation.

Molecular information may also change during treatment. Circulating tumor DNA (ctDNA) provides a minimally invasive approach to reassessing tumor biology and may complement tissue profiling when archival tissue no longer reflects the current molecular landscape or when repeat biopsy is not feasible [2,40]. ctDNA may capture spatial and temporal heterogeneity that is not represented by a single tissue specimen [6,41].

Anti-EGFR rechallenge provides one of the clearest clinical examples of this dynamic approach. The decline of acquired resistance alterations after withdrawal of EGFR blockade provides a biological rationale for molecular reassessment, and prospective studies support ctDNA-based selection of patients without detectable resistance alterations for rechallenge [14,42,43]. However, broader longitudinal use of ctDNA to guide treatment changes remains less well established and should be distinguished from applications supported by prospective clinical evidence.

The practical value of molecular profiling also depends on access to testing, assay performance, timing, turnaround time, and whether patients remain fit enough to receive the treatment identified. Real-world studies can help characterize these aspects of implementation, but favorable outcomes in comprehensively profiled or molecularly matched populations require cautious interpretation. Such patients may be selected for better performance status, access to specialized centers, or greater opportunity to receive subsequent therapies.

Molecular profiling in later-line mCRC should therefore combine established baseline testing with selective reassessment when the result has a realistic potential to change treatment. The principal molecular tools and their role in later-line decision-making are summarized in Table 1.

### 3.3. Redefining Clinically Meaningful Outcomes Beyond Overall Survival

Overall survival (OS) remains the most robust endpoint for establishing efficacy in later-line mCRC, but it does not fully capture the clinical value of treatment in heavily pretreated patients. Disease control, progression-free survival (PFS), preservation of performance status (PS), health-related quality of life (HRQoL), and treatment tolerability may all contribute to meaningful benefit and influence whether patients remain eligible for subsequent therapies [2,3].

In chemorefractory disease, benefit is often achieved through disease stabilization rather than objective tumor shrinkage, making disease control particularly relevant for treatments with predominantly cytostatic activity [2,38]. Similarly, PFS can provide a pragmatic measure of the time during which disease remains controlled before another treatment decision becomes necessary [2].

Preserving PS and HRQoL becomes increasingly important as patients accumulate previous treatments and cumulative toxicity [38]. Prospective later-line studies have also shown that patient-reported outcomes can be collected in this setting; PRECONNECT, for example, assessed quality of life during treatment with trifluridine/tipiracil [49]. These measures complement, rather than replace, conventional efficacy endpoints.

From a longitudinal perspective, treatment tolerability also matters because excessive toxicity or clinical deterioration may compromise access to subsequent active therapies. Preserving the therapeutic trajectory—maintaining disease control while limiting toxicity and loss of fitness—is therefore a relevant consideration when planning later-line treatment [2].

RWE can complement trial data by describing treatment duration, dose modifications, reasons for discontinuation, toxicity management, and the feasibility of subsequent therapy in routine practice [3]. Such information helps place OS and PFS within the broader treatment continuum, although these real-world outcomes should be interpreted according to the quality and limitations of the underlying data.

### 3.4. Therapeutic Sequencing: From Drug Selection to Personalized Longitudinal Care

#### 3.4.1. Sequencing as a Trajectory Problem

Later-line sequencing is increasingly a longitudinal rather than a single-line decision. Each treatment may affect subsequent options through cumulative toxicity, evolving resistance, changes in performance status, or loss of organ reserve. Treatment selection should therefore take into account prior exposure and tolerability, residual fitness, disease kinetics and symptom burden, actionable molecular alterations, and patient goals [2].

Attrition is an important practical constraint. Not all patients who are candidates for third-line treatment will remain fit enough to receive subsequent therapy. Planning therefore needs to balance the immediate need for disease control against previous benefit, ongoing toxicity, and the likelihood of reaching another treatment line [2].

#### 3.4.2. Why Conventional RCTs Rarely Answer Sequencing Questions

Registration trials generally establish the efficacy and safety of an individual treatment but rarely compare complete therapeutic sequences. For example, prospective evidence does not establish whether regorafenib should routinely precede trifluridine/tipiracil or vice versa. Current guidelines therefore do not identify a universally preferred order and support individualized sequencing according to clinical context [3,50].

#### 3.4.3. Strategy Trials and Real-World Evidence

Strategy trials and observational studies address different aspects of this evidence gap. STRATEGIC-1 illustrates both the potential value and the methodological difficulty of prospectively testing multiline treatment strategies [51]. Real-world cohorts, in turn, can describe treatment duration, dose modification, toxicity-related discontinuation, and transition to subsequent therapy in populations that are often less selected than those enrolled in registration trials [52,53].

Comparative real-world sequencing analyses require particular caution. Patients who receive several successive therapies are selected by survival, fitness, and access to treatment, creating substantial potential for selection bias, treatment attrition, immortal-time bias, and time-dependent confounding. Observed differences between treatment sequences should therefore remain hypothesis-generating unless the study design and analytical methods adequately address these sources of bias.

#### 3.4.4. Molecularly Informed Sequencing

Molecular profiling adds another dimension to sequencing. When a validated actionable alteration is present, matched therapy may appropriately take priority over molecularly unselected later-line options, provided that the patient has not already received the corresponding treatment and remains clinically eligible [2,3].

Dynamic molecular reassessment may further influence selected treatment decisions. ctDNA is particularly relevant to anti-EGFR rechallenge, where reassessment of resistance alterations can refine patient selection [2,51]. This represents a clinically supported use of dynamic molecular information, but it should not be extrapolated to imply that broad ctDNA-driven adaptive sequencing has been validated across later-line mCRC.

#### 3.4.5. Toward Patient-Specific Sequencing

In practice, later-line sequencing is best approached as a flexible clinical framework rather than a fixed algorithm. Treatment choices need to be revisited as disease characteristics, patient fitness, cumulative toxicity, previous benefit, and molecular information change over time. The aim is not necessarily to identify a single optimal sequence for all patients, but to preserve access to effective treatments while avoiding choices that may unnecessarily compromise subsequent options.

RWE can inform this process by showing how treatments and sequences are actually delivered in routine care. Its contribution is strongest when describing feasibility, treatment delivery, and attrition; claims of comparative superiority between sequences require a higher level of methodological rigor. The main clinical and molecular determinants of later-line sequencing are summarized in Table 2.

## 4. Real-World Evidence Across Available Later-Line Therapies

The therapeutic landscape of metastatic colorectal cancer (mCRC) beyond second line has expanded considerably. Later-line treatment now includes molecularly unselected agents, combination strategies, anti-EGFR rechallenge, immunotherapy, and biomarker-directed therapies. These options differ in mechanism, toxicity profile, molecular requirements, and practical feasibility and should therefore be considered within the overall therapeutic continuum rather than as isolated treatments [2,3].

**Figure 3:**
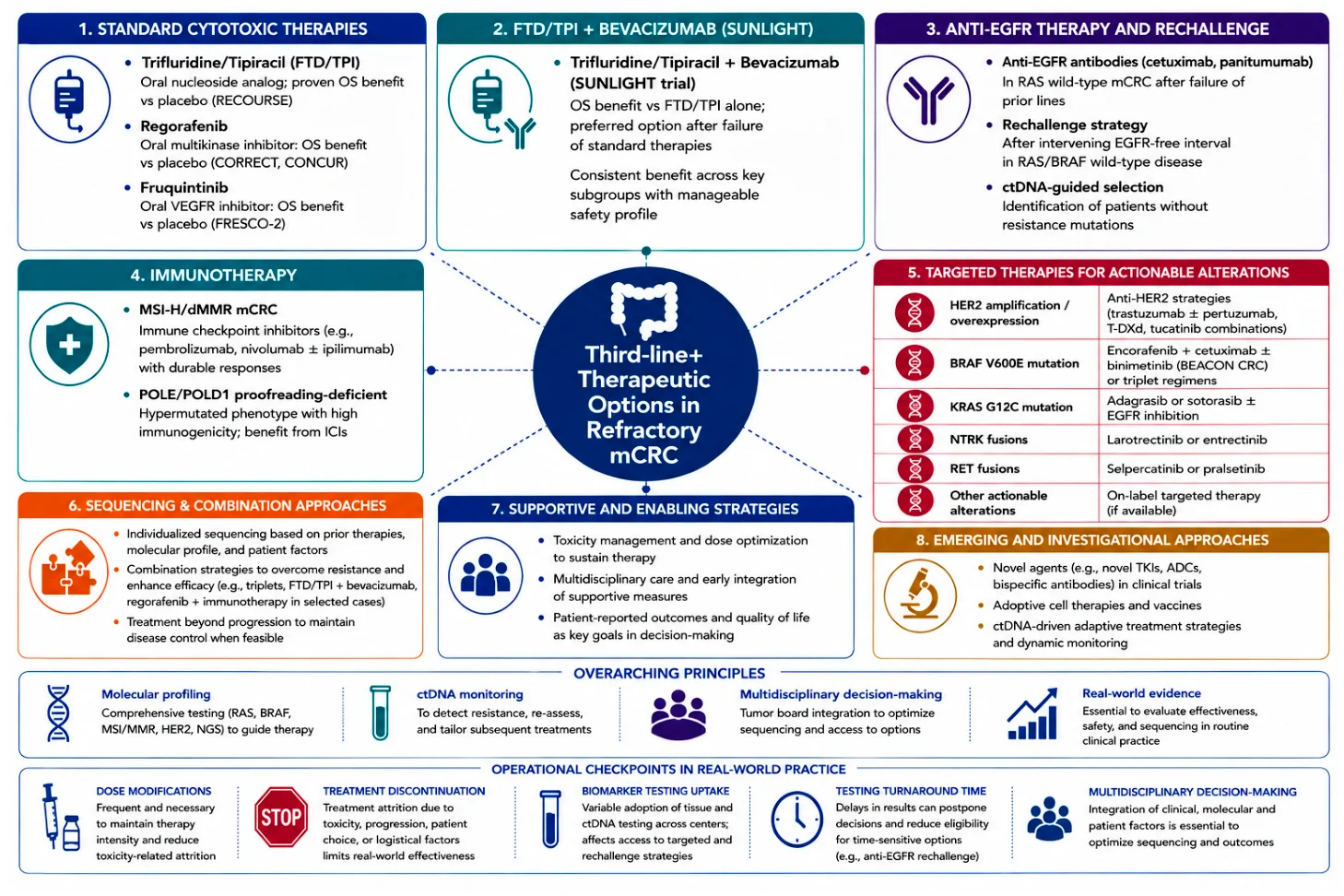
Therapeutic landscape of third-line and later metastatic colorectal cancer: from conventional later-line therapies to biomarker-driven precision oncology. Schematic overview of therapeutic strategies for third-line and later metastatic colorectal cancer (mCRC), integrating established later-line agents, antiangiogenic approaches, anti-EGFR rechallenge, immune checkpoint inhibition and biomarker-directed targeted therapies. Treatment selection and sequencing are increasingly individualized according to molecular profile, prior treatment exposure, residual fitness, disease burden, cumulative toxicity and patient-specific factors, with longitudinal ctDNA assessment and real-world evidence supporting adaptive precision-oncology strategies. Abbreviations: ADC, antibody–drug conjugate; ctDNA, circulating tumor DNA; EGFR, epidermal growth factor receptor; FTD/TPI, trifluridine/tipiracil; HER2, human epidermal growth factor receptor 2; ICI, immune checkpoint inhibitor; mCRC, metastatic colorectal cancer; MSI-H/dMMR, microsatellite instability-high/mismatch repair deficient; NGS, next-generation sequencing; OS, overall survival; T-DXd, trastuzumab deruxtecan; TKI, tyrosine kinase inhibitor; VEGFR, vascular endothelial growth factor receptor.

Pivotal randomized trials have established the efficacy and safety of regorafenib, trifluridine/tipiracil (FTD/TPI), and fruquintinib in refractory mCRC, while prospective studies have supported biomarker-directed strategies for selected molecular subgroups [2]. However, randomized trials provide less information on comparative sequencing, treatment delivery, dose modification, and outcomes in older, frail, or comorbid patients who are frequently encountered in routine practice [2,3]. The current later-line therapeutic landscape and the main clinical and molecular factors influencing treatment selection are summarized in Figure 3.

RWE can complement this evidence by describing effectiveness and safety in less-selected populations, treatment delivery and dose optimization, and patterns of treatment sequencing in routine care [3,38]. Its contribution varies according to the treatment and the question being addressed. For regorafenib, observational studies have been particularly informative regarding dosing and toxicity management; for FTD/TPI-based treatment, they have extended information on tolerability and use in vulnerable populations [54]; and observational datasets have explored sequencing between later-line agents when prospective comparisons are unavailable [55].

These findings should nevertheless be interpreted according to study design. RWE is well suited to describing implementation, feasibility, and safety in routine practice, whereas comparative treatment or sequencing analyses remain vulnerable to selection bias, attrition, and residual or time-dependent confounding. The principal areas in which RWE complements randomized evidence in later-line mCRC are summarized in Table 3.

### 4.1. Regorafenib: The Paradigm of Real-World Evidence in Later-Line mCRC

#### 4.1.1. From Registration Trials to Real-World Practice

The phase III CORRECT trial established the efficacy of regorafenib in chemorefractory mCRC, demonstrating an improvement in overall survival (OS; 6.4 vs. 5.0 months) and progression-free survival compared with placebo, with disease stabilization accounting for most of the clinical activity [7]. CONCUR subsequently confirmed a survival benefit in an Asian population with different prior-treatment patterns [8].

Translation into routine practice, however, highlighted the importance of treatment delivery. At the labelled starting dose of 160 mg/day, early toxicities—including hand–foot skin reaction (HFSR), fatigue, hypertension, diarrhea, and less commonly serious hepatotoxicity—frequently required dose modification [3]. This provided a clear setting in which RWE could complement registration trials.

CORRELATE, a prospective observational study including 1037 patients, reported effectiveness broadly consistent with the pivotal trials while showing considerable variability in starting dose and subsequent dose modification [20]. CONSIGN, a large phase IIIb study including 2872 patients, similarly confirmed the expected safety profile and showed that adverse events could often be managed through active monitoring and dose adjustment [21]. REBECCA further identified poorer performance status and greater metastatic burden among factors associated with worse outcome [61], while a large Japanese post-marketing study emphasized that adverse events occur predominantly early in treatment and require close monitoring during the first cycles [62].

#### 4.1.2. Dose Optimization and Treatment Delivery

Dose optimization represents one of the clearest examples of how post-registration evidence changed the practical use of regorafenib. The 160-mg starting dose used in CORRECT and CONCUR may be difficult to sustain in some patients because toxicity is concentrated early in treatment [7,8]. In the randomized phase II ReDOS study, a strategy starting at 80 mg/day with weekly escalation to 160 mg/day according to tolerability resulted in a higher proportion of patients reaching cycle 3 than standard-dose initiation (43% vs. 26%) [28]. This approach was subsequently incorporated into NCCN guidance as an alternative dosing strategy [3].

REARRANGE provided further prospective evidence on alternative induction strategies [63], while a US claims analysis of 703 patients showed increasing adoption of flexible dosing after publication of ReDOS and its incorporation into clinical guidance [64]. Taken together, these data support individualized treatment delivery rather than assuming that every patient should follow the same starting-dose strategy.

#### 4.1.3. Patient Selection and Prognostic Factors

Across real-world studies, performance status has consistently emerged as an important determinant of treatment exposure and outcome, supporting the use of regorafenib while patients retain adequate clinical fitness [2,61,62]. Higher metastatic burden and liver involvement have also been associated with poorer prognosis, whereas treatment-emergent findings such as early HFSR should be regarded as exploratory rather than validated predictive biomarkers [61,62].

Older age alone should not exclude treatment. Geriatric oncology guidance emphasizes functional status, comorbidity, and tolerability rather than chronological age as the basis for treatment decisions [58]. More broadly, the available evidence supports a distinction between prognostic factors, which may identify patients with different expected outcomes, and predictive biomarkers, for which no validated marker currently guides regorafenib selection [61,62,65].

#### 4.1.4. Regorafenib Within Later-Line Sequencing

With several active later-line treatments now available, the position of regorafenib depends increasingly on the overall therapeutic trajectory. No randomized evidence establishes a preferred sequence between regorafenib and FTD/TPI, and treatment order should therefore be individualized according to residual fitness, toxicity, disease characteristics, previous treatment, molecularly defined alternatives, and patient priorities [2,3].

Observational studies have attempted to address this evidence gap. REGOTAS used propensity-score methods to compare regorafenib with FTD/TPI in routine practice [66]. ReTrITA subsequently evaluated treatment sequences directly and reported better survival outcomes for regorafenib followed by FTD/TPI than for the reverse sequence [30], while nationwide database evidence from Taiwan also suggested longer OS with this order [55]. SEQRT2 and other comparative real-world cohorts provide additional information on sequential treatment delivery [57,67]. These findings are clinically informative but cannot establish sequence superiority because treatment allocation, attrition, residual confounding, and survival to subsequent lines may influence the observed associations. They should therefore be regarded as hypothesis-generating rather than equivalent to randomized sequence comparisons.

#### 4.1.5. Current Perspective

No validated predictive biomarker currently identifies patients who are specifically more likely to benefit from regorafenib [65]. Combination approaches, including regorafenib with immune checkpoint inhibitors, remain investigational. Meta-analytic and observational evidence is derived largely from non-randomized studies and does not establish a routine role for these combinations [68,69].

The most established contribution of precision to regorafenib treatment therefore remains practical rather than molecular: appropriate patient selection, individualized dosing, early toxicity management, and integration within the later-line therapeutic sequence [2,3]. In this setting, RWE has complemented randomized evidence primarily by showing how regorafenib can be delivered in heterogeneous routine-care populations rather than by establishing new treatment effects.

The key randomized and real-world studies that have shaped the clinical implementation of regorafenib are summarized in Table 4.

### 4.2. Trifluridine/Tipiracil: Real-World Evidence and the Evolution of Precision Later-Line Therapy

#### 4.2.1. From Recourse to Contemporary Clinical Practice

The phase III RECOURSE trial established the efficacy of FTD/TPI in refractory mCRC, with improvements in overall survival (OS; 7.1 vs. 5.3 months) and progression-free survival compared with placebo [9]. Myelosuppression, particularly neutropenia and leukopenia, was the main toxicity. FTD/TPI also delayed deterioration to ECOG performance status ≥ 2 (5.7 vs. 4.0 months), an important consideration in a setting in which preservation of fitness may influence access to subsequent treatment [9].

The predominantly hematologic toxicity profile of FTD/TPI differs from the non-hematologic toxicities commonly associated with multikinase inhibitors and has practical implications for treatment selection and monitoring [3]. Subsequent real-world and post-authorization studies extended the evidence beyond RECOURSE by examining effectiveness and tolerability in less-selected populations, including older patients, as well as dose modification, treatment exposure, and hematologic toxicity in routine practice [38,58].

#### 4.2.2. Chemotherapy-Induced Neutropenia as a Pharmacodynamic Marker

Chemotherapy-induced neutropenia (CIN) has emerged as one of the most consistent treatment-emergent correlates of outcome during FTD/TPI therapy. In a pharmacokinetic–pharmacodynamic analysis of RECOURSE and J003, greater trifluridine exposure was associated with an increased likelihood of neutropenia, while early CIN was associated with improved OS and PFS [59].

These findings support CIN as a potential pharmacodynamic marker of treatment exposure rather than a validated predictive biomarker. Its occurrence may provide information on drug exposure under standard dosing, but absence of neutropenia should not be used to justify unsafe dose escalation [59]. Accordingly, CIN should be distinguished from predictive biomarkers used for treatment selection.

Other potential markers remain exploratory. A large analysis integrating real-world data with RECOURSE suggested that the magnitude of FTD/TPI benefit may differ according to specific *KRAS* variants, including *KRAS G12C*, but these findings require prospective validation before influencing treatment selection [70].

#### 4.2.3. FTD/TPI Plus Bevacizumab and Treatment Delivery

The phase III SUNLIGHT trial changed the later-line treatment framework by demonstrating that FTD/TPI plus bevacizumab improved OS (10.8 vs. 7.5 months) and PFS (5.6 vs. 2.4 months) compared with FTD/TPI alone [71]. The combination is now preferentially recommended over FTD/TPI monotherapy when appropriate and available [2,3].

RWE is beginning to clarify how the combination performs outside the population selected for randomized trials. The multicenter retrospective WJOG14520G (TWILIGHT) study evaluated FTD/TPI plus bevacizumab in vulnerable, previously treated patients considered unsuitable for intensive therapy, supporting its feasibility while emphasizing the need for hematologic monitoring [54]. A contemporary multicenter cohort also compared FTD/TPI plus bevacizumab with FTD/TPI or regorafenib in routine practice [72].

Treatment delivery remains clinically relevant. TALLISUR prospectively evaluated FTD/TPI in a broad practice setting and incorporated health-related quality-of-life assessment, providing information not collected in RECOURSE [73]. Alternative schedules are also being investigated to reduce myelosuppression while maintaining treatment exposure, although these approaches should not yet be considered equivalent to established dosing strategies without adequate prospective evidence [74,75].

#### 4.2.4. FTD/TPI Within Later-Line Sequencing

The optimal position of FTD/TPI relative to regorafenib and fruquintinib remains uncertain. Current guidelines support individualized sequencing according to patient fitness, disease burden and need for response, previous treatment benefit, and cumulative toxicity; no prospective evidence establishes a universally preferred order between FTD/TPI and regorafenib [2,3].

Observational comparative studies provide useful but non-definitive information, including US and multicenter comparisons of FTD/TPI-based treatment and regorafenib [67,72,76]. As with other observational sequencing studies, however, treatment selection, attrition, and residual or time-dependent confounding limit causal interpretation. Such findings should therefore be regarded as hypothesis-generating rather than evidence of sequence superiority.

The increasing availability of biomarker-directed therapies further influences treatment order. For patients with actionable molecular alterations, targeted or immune-based strategies may appropriately modify the timing of molecularly unselected agents such as FTD/TPI [2].

#### 4.2.5. Current Perspective

FTD/TPI-based treatment illustrates how randomized and real-world evidence can address different clinical questions. RECOURSE established the efficacy of FTD/TPI monotherapy and SUNLIGHT established the benefit of adding bevacizumab, whereas RWE has contributed mainly to understanding treatment delivery, toxicity, use in less-selected populations, and sequencing. CIN is an informative pharmacodynamic correlate but not a validated predictive biomarker, while *KRAS* subtype and ctDNA-based approaches remain investigational [59,70,77]. The key evidence shaping FTD/TPI-based later-line treatment is summarized in Table 5.

### 4.3. Fruquintinib: Expanding the Antiangiogenic Strategy in Later-Line mCRC

#### 4.3.1. From Fresco to Global Clinical Practice

Following the initial FRESCO study in China, the international phase III FRESCO-2 trial established the efficacy of fruquintinib in a contemporary refractory population previously exposed to standard therapies, including regorafenib and/or FTD/TPI when available [10]. FRESCO-2 demonstrated significant improvements in OS and PFS compared with placebo, with a safety profile mainly characterized by VEGF-pathway toxicities, including hypertension, proteinuria, and hand–foot skin reaction [10].

Fruquintinib has consequently been incorporated into later-line treatment algorithms. Current guidance positions it among the options for molecularly unselected patients after progression on established therapies, with treatment order individualized according to previous exposure, residual fitness, disease characteristics, and ongoing toxicity [2,10].

#### 4.3.2. Emerging Real-World Evidence

Compared with regorafenib and FTD/TPI, the real-world evidence base for fruquintinib remains less mature. Early observational experience has provided information on effectiveness and toxicity outside registration trials, but its contribution to treatment selection and sequencing is still evolving [78].

In routine practice, RWE may be particularly useful for characterizing dose interruptions and reductions, treatment discontinuation, and tolerability in patients who are older, have relevant comorbidities, or have borderline performance status [2]. These data are important for understanding treatment feasibility but should not be interpreted as evidence of comparative superiority over other later-line agents.

No validated predictive biomarker currently guides selection for fruquintinib. Associations between clinical characteristics and outcome should therefore be regarded as prognostic unless a treatment-specific predictive effect is demonstrated [2].

#### 4.3.3. Positioning Fruquintinib Within the Therapeutic Continuum

No randomized trial has directly compared fruquintinib with regorafenib or FTD/TPI, and no prospective evidence establishes an optimal sequence among these agents. Its position should therefore be individualized according to previous treatment, molecularly defined alternatives, disease burden, residual toxicity, and patient fitness [2,10].

The different toxicity profiles of available later-line agents may help guide treatment selection. In particular, hypertension, proteinuria, and hand–foot skin reaction with fruquintinib need to be considered alongside residual toxicities from previous treatments, including impaired marrow reserve after cytotoxic therapy or persistent toxicity from multikinase inhibitors [2,3].

Observational studies may eventually clarify the relative feasibility of different sequences, but comparative analyses require careful consideration of selection bias, treatment attrition, and time-dependent confounding. At present, RWE is therefore more informative for treatment delivery and external validity than for establishing a preferred sequence.

#### 4.3.4. Current Perspective

Fruquintinib is supported by randomized phase III evidence as a later-line treatment, whereas its optimal implementation in routine practice remains an evolving area of RWE. Further observational research should focus on treatment delivery, tolerability in less-selected populations, attrition, and positioning within contemporary treatment sequences [2,22,78]. Combination strategies with immune checkpoint inhibitors and ctDNA-based monitoring during antiangiogenic treatment remain investigational and should not currently influence routine treatment selection [2,79].

### 4.4. Anti-EGFR Rechallenge: A Paradigm of ctDNA-Guided Precision Oncology

#### 4.4.1. From Clinical Observation to Molecular Selection

The biological rationale for rechallenge derives from the dynamic nature of acquired anti-EGFR resistance. Under EGFR blockade, resistant subclones—commonly involving RAS, *BRAF*, and EGFR ectodomain alterations—may expand, whereas after treatment withdrawal their relative abundance can decline [44]. This clonal decay provides a mechanistic basis for renewed anti-EGFR sensitivity after an EGFR-free interval.

ctDNA has made this concept clinically accessible by allowing repeated molecular reassessment without the need for serial tissue biopsies [2,14]. Rechallenge has therefore evolved from an empirically selected strategy based mainly on previous response and treatment-free interval toward a molecularly selected approach in which the current resistance profile contributes to treatment eligibility [14].

#### 4.4.2. Clinical Evidence for ctDNA-Guided Rechallenge

The phase II CRICKET study provided an important proof of concept. In patients rechallenged with cetuximab plus irinotecan, absence of detectable RAS resistance alterations in baseline ctDNA was associated with longer PFS, supporting molecular selection beyond clinical criteria alone [45].

CHRONOS subsequently prospectively incorporated ctDNA into patient selection, using a “zero-mutation” strategy for RAS, *BRAF*, and EGFR ectodomain resistance alterations before chemotherapy-free panitumumab rechallenge [14]. This represented an important transition from retrospective biomarker association to prospective molecular selection.

PARERE further addressed the position of anti-EGFR rechallenge within later-line sequencing by comparing treatment strategies involving panitumumab and regorafenib in ctDNA RAS/*BRAF* wild-type patients [46]. VELO and CAVE-2 further extended randomized evidence for rechallenge strategies in molecularly selected populations [42,43]. In addition, an individual patient-data pooled analysis of Italian rechallenge studies, including CRICKET and CHRONOS, supported clinically meaningful activity in ctDNA RAS/*BRAF* wild-type patients while suggesting that broader negative molecular selection may further refine patient identification [47].

Together, these studies support ctDNA-based molecular reassessment before anti-EGFR rechallenge. They should not, however, be interpreted as validation of unrestricted longitudinal ctDNA-guided treatment adaptation across later-line mCRC.

#### 4.4.3. Real-World Implementation and Current Limitations

The contribution of RWE in this setting lies particularly in implementation. Routine use of ctDNA depends on assay availability, turnaround time, timing of sampling, analytical sensitivity, interpretation of low-frequency variants, and integration of results with competing treatment options [2,40].

Important biological and methodological limitations also remain. ctDNA detection is influenced by tumor shedding and disease distribution, while assays differ in coverage and sensitivity. A negative result therefore does not invariably exclude resistant subclones. Repeated-testing cohorts may also be selected for patients with better performance status, access to specialized centers, and sufficient survival to undergo serial molecular assessment.

Translational and real-world studies can help characterize resistance alterations beyond RAS/*BRAF* and EGFR ectodomain mutations and may inform broader “negative hyperselection” approaches [2,6]. These strategies remain an area of investigation rather than a uniformly validated standard.

#### 4.4.4. Current Perspective

At present, the strongest clinical application of longitudinal ctDNA in later-line mCRC is molecular reassessment for anti-EGFR rechallenge. More extensive adaptive strategies based on serial ctDNA kinetics remain investigational.

RASINTRO provides prospective evidence that baseline ctDNA RAS/*BRAF* status and early changes in ctDNA during rechallenge are associated with outcome [48]. Whether serial quantitative ctDNA monitoring should be used to modify treatment in real time, however, requires further prospective validation. Questions regarding optimal testing intervals, low-level or polyclonal resistance, assay standardization, and the value of expanded resistance panels also remain unresolved [6,44].

The principal evidence supporting the transition from empirically selected to ctDNA-guided anti-EGFR rechallenge is summarized in Table 6.

### 4.5. Immunotherapy in MSI-H/dMMR Metastatic Colorectal Cancer: Real-World Evidence Beyond Clinical Trials

#### 4.5.1. Established Biomarker-Selected Immunotherapy

Immune checkpoint inhibition is an established treatment for MSI-H/dMMR mCRC and should be clearly distinguished from investigational immunotherapy strategies in pMMR/MSS disease. In patients with MSI-H/dMMR tumors who have not previously received immunotherapy, PD-1 blockade represents an established therapeutic option, including in previously treated disease [2,80].

The marked sensitivity of MSI-H/dMMR tumors to immune checkpoint inhibition has established MMR/MSI status as a predictive biomarker for immunotherapy in mCRC [2,80]. Although immune checkpoint inhibitors have increasingly moved to earlier treatment lines, later-line treatment remains relevant for patients who are ICI-naïve at progression.

RWE complements prospective trial evidence primarily by addressing external validity. Patients treated in routine practice may be older, have relevant comorbidities or poorer performance status, and may differ from those enrolled in registration trials. Observational cohorts and systematic reviews of real-world studies can therefore provide information on treatment exposure, durability of benefit, toxicity management, and outcomes in these less-selected populations [22,81].

Rare *POLE*/*POLD1* proofreading-domain alterations represent a distinct hypermutated subgroup in which substantial immunotherapy activity has also been reported, including in selected MSS tumors. These cases require appropriate molecular classification and should not be generalized to unselected pMMR/MSS disease [2,3,82].

#### 4.5.2. Real-World Questions After Immunotherapy Initiation

Several clinically relevant questions remain incompletely addressed by prospective trials. Pseudoprogression is uncommon in CRC but may complicate response assessment in selected patients, requiring integration of imaging findings with clinical status before treatment discontinuation [83].

The optimal duration of immunotherapy in patients with deep and durable responses is also uncertain. Prospective evidence defining when treatment can safely be discontinued remains limited, making registries and observational cohorts potentially useful for describing outcomes after elective discontinuation [2,3].

Management after progression on immune checkpoint inhibition represents another evidence gap. In the absence of a definitive prospective standard, subsequent treatment should be individualized according to previous therapy, molecular profile, disease pattern, and available later-line options [2,80]. RWE can describe these treatment trajectories but cannot establish the superiority of one post-ICI sequence over another.

#### 4.5.3. Current Perspective

The distinction between established and investigational immunotherapy is particularly important in later-line mCRC. For MSI-H/dMMR, and in selected POLE/POLD1 proofreading-deficient tumors, immune checkpoint inhibition is supported by biomarker-defined clinical evidence [2,3,80,82,84]. By contrast, immune checkpoint inhibitor monotherapy is not an established treatment for unselected pMMR/MSS mCRC [2,80].

Combination approaches designed to overcome immune resistance in pMMR/MSS disease—including chemo-immunotherapy and antiangiogenic/TKI–ICI strategies—remain investigational and should not be presented as routine later-line treatment [68,79,85,86]. Similarly, longitudinal ctDNA monitoring for determining immunotherapy duration or directing post-ICI treatment changes remains investigational [2].

In this setting, the most established contribution of RWE is therefore to characterize external validity, treatment duration, toxicity, and post-treatment trajectories, rather than to define new immunotherapy indications or treatment sequences.

### 4.6. HER2-Targeted Therapy: Precision Treatment and Real-World Implementation

#### 4.6.1. Clinical Development of HER2-Targeted Therapy

HER2 (*ERBB2*) amplification/overexpression defines an actionable molecular subgroup of mCRC for which several targeted strategies have been developed. In later-line disease, treatment selection depends not only on HER2 status but also on RAS status, level of HER2 expression, previous anti-HER2 exposure, molecular heterogeneity, and treatment-specific toxicity [2,3,87].

Early prospective studies established HER2 as a therapeutic target in refractory mCRC. HERACLES provided proof of concept for dual HER2 blockade with trastuzumab plus lapatinib [88], while MyPathway demonstrated activity with trastuzumab plus pertuzumab in previously treated HER2-amplified disease [3].

More recently, MOUNTAINEER established tucatinib plus trastuzumab as a chemotherapy-free strategy in HER2-positive, RAS wild-type mCRC after standard chemotherapy [3,89]. In parallel, DESTINY-CRC01 and DESTINY-CRC02 demonstrated clinically meaningful activity with trastuzumab deruxtecan (T-DXd), with benefit enriched in HER2-high tumors, particularly IHC 3+ disease [90,91].

The evidence base remains largely derived from phase II and single-arm studies with different eligibility and HER2 testing criteria. Consequently, the optimal sequence among tucatinib–trastuzumab, trastuzumab-based dual blockade, and T-DXd has not been established prospectively [2].

#### 4.6.2. Biomarker Implementation and the Role of RWE

HER2 testing is a central implementation issue in routine practice. HER2 status may be assessed using IHC/ISH and increasingly through tissue- or blood-based genomic testing, but amplification, protein expression, and genomic detection should not be regarded as interchangeable measures without considering the assay and clinical context [2,92].

ctDNA may facilitate molecular assessment when tissue is limited and can provide information on tumor heterogeneity and resistance evolution. However, a negative plasma result may be less informative in low-shedding disease and should be interpreted together with available tissue findings. MOUNTAINEER also provided evidence of molecular heterogeneity at progression, including loss of detectable *ERBB2* amplification in some patients [89].

RAS status further influences treatment selection. Tucatinib–trastuzumab was developed in RAS wild-type disease, whereas T-DXd has shown activity in HER2-high tumors across a broader molecular context. HER2 expression level is also clinically relevant, since activity with T-DXd has been substantially greater in HER2-high than in HER2-low disease [2,90,91].

Safety is another area in which routine-care evidence is important. Interstitial lung disease/pneumonitis is a clinically relevant toxicity of T-DXd requiring proactive surveillance and early management [93]. DESTINY-CRC02 further informed dose selection, supporting 5.4 mg/kg as the preferred dose in this setting [3,91].

RWE can therefore complement prospective evidence by characterizing testing uptake, access to matched therapy, assay concordance, toxicity management, and treatment sequencing in less-selected populations [22,92]. Comparative effectiveness between HER2-directed platforms, however, remains uncertain and should not be inferred from non-randomized comparisons.

#### 4.6.3. Current Perspective

HER2-directed therapy illustrates the importance of distinguishing an established predictive biomarker from emerging approaches to longitudinal molecular monitoring. HER2 amplification/overexpression supports treatment selection, but quantitative expression, *ERBB2* copy number, RAS/RAF co-alterations, and spatial or temporal heterogeneity may further influence response and resistance [89,94,95].

Reassessment after progression may help characterize loss of HER2 dependence or emerging bypass mechanisms, but using serial ctDNA or other molecular changes to direct sequential HER2 therapy remains investigational [2,89,95]. Registries and multicenter observational cohorts may be particularly valuable for evaluating real-world testing, sequencing, and uncommon but clinically important toxicities in this relatively rare molecular subgroup [92,93].

### 4.7. BRAF V600E-Mutated mCRC: Targeted Therapy and Real-World Implementation

#### 4.7.1. From Beacon to Routine Practice

BRAF V600E identifies a biologically distinct subgroup of mCRC for which combined BRAF and EGFR inhibition has become an established treatment strategy after previous therapy. The rationale for combined blockade derives from adaptive EGFR reactivation during BRAF inhibition, which limits the activity of BRAF inhibitor monotherapy in colorectal cancer [12,96].

The phase III BEACON CRC trial established encorafenib plus cetuximab as a standard treatment for previously treated *BRAF V600E*-mutated mCRC. In the updated analysis, median OS was 9.3 months with the doublet compared with 5.9 months in the control group (HR 0.61) [97]. The addition of binimetinib increased response numerically but did not improve survival over the doublet and added MEK-related toxicity [2,3].

Current guidelines therefore support encorafenib plus an anti-EGFR antibody in previously treated *BRAF V600E*-mutated disease [2,3,98]. However, BEACON predominantly enrolled patients with ECOG performance status 0–1, leaving clinically relevant questions regarding treatment delivery in frailer populations and optimal positioning relative to other later-line therapies [2].

#### 4.7.2. Real-World Evidence, Sequencing, and Resistance

Post-approval RWE has mainly addressed the external validity and implementation of BRAF-targeted therapy. The prospective nationwide BEETS (JACCRO CC-18) study evaluated encorafenib–cetuximab ± binimetinib in routine practice, providing information on treatment delivery and outcomes in a population less selected than that enrolled in BEACON [99].

This is particularly relevant because patients with refractory *BRAF V600E*-mutated disease may experience rapid clinical deterioration, making treatment timing and preservation of performance status important determinants of whether targeted therapy can actually be delivered [2]. RWE can describe these patterns and treatment attrition, but it cannot establish an optimal sequence between *BRAF*-targeted therapy and other later-line options in the absence of randomized comparative evidence [22].

Resistance after *BRAF*/EGFR blockade is frequently heterogeneous. Molecular profiling from BEACON using tissue and ctDNA identified polyclonal acquired resistance, including alterations involving *KRAS*, *NRAS*, *MAP2K1*, and *MET* amplification [100]. These findings help explain why progression after targeted therapy cannot usually be addressed through a single uniform molecular strategy.

In routine practice, toxicity management also remains important. Observational experience supports early supportive care and dose modification when required to maintain treatment exposure, particularly in patients at risk of rapid clinical decline [97,99].

#### 4.7.3. Current Perspective

For *BRAF V600E*-mutated mCRC, the distinction between established and investigational precision strategies is important. Encorafenib plus anti-EGFR therapy is supported by randomized evidence, whereas ctDNA-guided adaptation after progression remains investigational [2,97,100].

Serial ctDNA assessment may provide information on early molecular response and emerging resistance during *BRAF*/EGFR inhibition, but its role in directing treatment changes has not been prospectively established [101]. Similarly, combinations incorporating immune checkpoint blockade or additional MAPK-pathway inhibition remain investigational in this setting and should not be considered routine later-line strategies [102].

### 4.8. KRAS G12C and Rare Actionable Alterations in Refractory mCRC

#### 4.8.1. *KRAS G12C*-Targeted Therapy

*KRAS G12C* has emerged as an actionable molecular alteration in mCRC, although effective targeting in colorectal cancer generally requires concomitant EGFR inhibition because adaptive EGFR signaling limits the activity of *KRAS G12C* inhibitor monotherapy [103,104].

The phase III CodeBreaK 300 trial established the clinical activity of combined *KRAS G12C* and EGFR inhibition in chemorefractory disease. Sotorasib 960 mg plus panitumumab improved median PFS compared with standard later-line therapy (5.6 vs. 2.2 months; HR 0.49) [103]. This randomized evidence directly supports combined pathway inhibition in previously treated *KRAS G12C*-mutated mCRC.

Adagrasib plus cetuximab has also demonstrated clinically meaningful activity in heavily pretreated patients. In KRYSTAL-1, the combination achieved an objective response rate of 34.0%, with median PFS of 6.9 months and median OS of 15.9 months [105]. Together, these studies support EGFR co-inhibition as a central component of *KRAS G12C*-targeted treatment in colorectal cancer [103,104,105].

The principal role of RWE in this setting is therefore not to establish treatment efficacy, but to clarify implementation in routine practice, including timely molecular testing, access to matched therapy, treatment delivery, toxicity management, and subsequent treatment patterns [2].

#### 4.8.2. Resistance and Post-Progression Management

Acquired resistance to *KRAS G12C* inhibition is molecularly heterogeneous and may involve reactivation of RAS–MAPK signaling and other bypass mechanisms [104,106]. Molecular reassessment, including ctDNA, may therefore help characterize resistance after progression and identify eligibility for clinical trials.

However, using serial ctDNA to direct treatment changes after *KRAS G12C*-targeted therapy remains investigational. Similarly, the optimal sequence of *KRAS G12C* inhibitors relative to other later-line therapies has not been established prospectively. RWE may help describe these treatment trajectories, but observational sequencing analyses should remain hypothesis-generating.

#### 4.8.3. Rare Actionable Alterations

Broad molecular profiling may also identify uncommon actionable alterations, including NTRK and *RET* fusions. Although individually rare in mCRC, these alterations may provide access to tumor-agnostic targeted therapies when detected [2]. Larotrectinib and other TRK-directed therapies have demonstrated activity across TRK fusion-positive solid tumors [107].

For these rare molecular subgroups, the contribution of RWE is particularly relevant because CRC-specific randomized trials are often impractical. Molecular registries and multicenter observational cohorts can complement basket-trial evidence by documenting testing pathways, access, treatment duration, toxicity, and resistance in routine practice. These data should complement rather than substitute for the prospective evidence supporting individual targeted therapies.

The main biomarker-driven later-line strategies and their implementation priorities are summarized in Table 7.

## 5. Future Directions: From Real-World Evidence to Adaptive Precision Oncology

Later-line mCRC is increasingly characterized by multiple therapeutic options, molecularly defined subgroups, and substantial attrition across successive treatments. Future progress will therefore depend not only on the development of new therapies but also on improving how available treatments, molecular information, and real-world data are integrated over time [2,60].

### 5.1. Dynamic Molecular Reassessment and Therapeutic Sequencing

Baseline molecular profiling remains essential, but tumor biology may evolve under therapeutic pressure. ctDNA offers a minimally invasive approach to molecular reassessment, particularly when a treatment transition is being considered. Its most clinically supported later-line application is currently the selection of patients for anti-EGFR rechallenge, whereas broader use of serial ctDNA to direct treatment changes remains investigational [2,6].

Important limitations include low tumor fraction, site-dependent DNA shedding, assay heterogeneity, and differences in reporting thresholds, all of which may affect the interpretation of negative or low-level findings [41,60]. Prospective validation and greater assay standardization will therefore be required before longitudinal ctDNA-guided adaptation can be generalized beyond specific clinical settings.

At the same time, the expanding number of later-line options makes therapeutic sequencing an increasingly important research question. Future RWE studies should move beyond descriptive treatment patterns and use designs capable of addressing selection bias, treatment attrition, immortal-time bias, and time-dependent confounding [60]. Clinically relevant endpoints should also capture treatment feasibility and cumulative benefit rather than focusing exclusively on outcomes from an individual treatment line.

**Figure 4:**
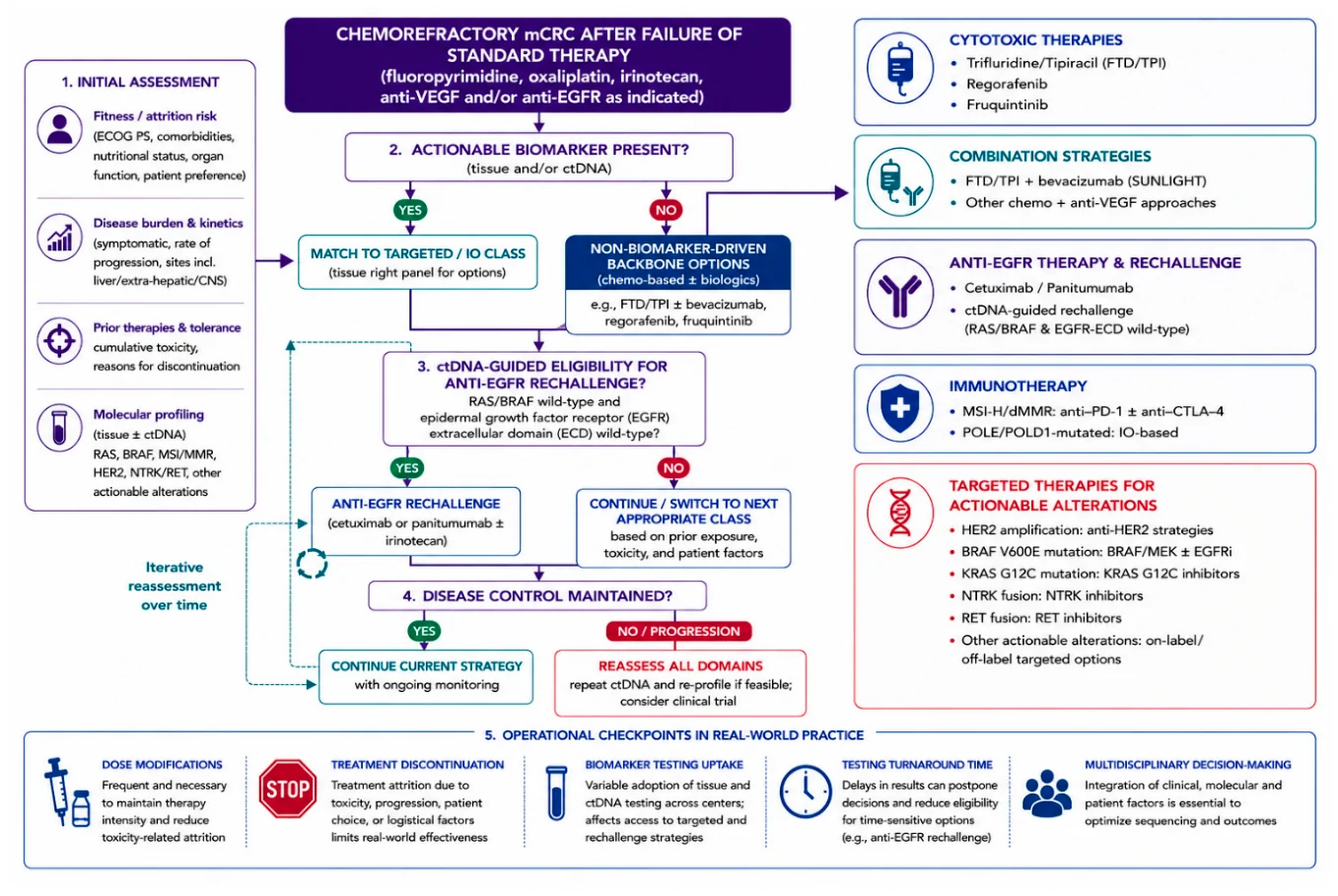
Third-line+ treatment sequencing in refractory metastatic colorectal cancer: an adaptive model integrating biomarkers and real-world delivery constraints. Conceptual scheme of an adaptive sequencing pathway in chemorefractory mCRC integrating fitness/attrition risk, disease kinetics, actionable biomarkers and ctDNA-guided eligibility (including anti-EGFR rechallenge). Operational checkpoints that commonly drive real-world feasibility (dose modifications, treatment discontinuation and biomarker testing uptake/turnaround time) are highlighted. Solid arrows indicate the main treatment decision pathway, whereas dashed arrows indicate iterative clinical and molecular reassessment over time. Abbreviations: *BRAF*, *B-Raf* proto-oncogene; CNS, central nervous system; ctDNA, circulating tumor DNA; ECD, extracellular domain; ECOG PS, Eastern Cooperative Oncology Group performance status; EGFR, epidermal growth factor receptor; EGFRi, epidermal growth factor receptor inhibitor; FTD/TPI, trifluridine/tipiracil; HER2, human epidermal growth factor receptor 2; IO, immuno-oncology; mCRC, metastatic colorectal cancer; MSI-H/dMMR, microsatellite instability-high/mismatch repair deficient; NTRK, neurotrophic tyrosine receptor kinase; PD-1, programmed cell death protein 1; POLE/POLD1, DNA polymerase epsilon/delta 1; *RAS*, rat sarcoma virus oncogene homolog; *RET*, rearranged during transfection; VEGF, vascular endothelial growth factor.

The clinical, molecular, and treatment-delivery factors that may contribute to this longitudinal approach are summarized in Figure 4.

### 5.2. Molecular Tumor Boards, Registries, and Rare Molecular Subgroups

The growing complexity of molecular testing creates an implementation challenge as well as a therapeutic opportunity. Molecular Tumor Boards (MTBs) can support interpretation of genomic and ctDNA findings, integration with previous treatment and patient fitness, and identification of matched therapies or clinical trials [2].

Their potential value is particularly relevant for uncommon actionable alterations, for which CRC-specific randomized trials may be difficult to conduct. Harmonized registries and multicenter real-world datasets can complement prospective and basket-trial evidence by characterizing testing uptake, access to treatment, toxicity, duration of benefit, and resistance in routine practice [2,60].

The timing of molecular testing is also important. Actionable alterations are clinically relevant only if they are identified while patients remain sufficiently fit to receive matched treatment. RWE can help quantify where testing delays, limited access, or treatment attrition result in missed therapeutic opportunities.

### 5.3. Data Integration and Decision Support

As clinical, molecular, imaging, and longitudinal data become increasingly complex, computational approaches may eventually assist treatment selection and sequencing. Artificial intelligence and machine-learning models have been proposed as tools for integrating multimodal information and estimating treatment benefit or toxicity [108].

However, their role in refractory mCRC remains investigational. Predictive performance alone does not establish clinical utility, and AI-based decision support would require standardized data, external validation, transparent methodology, appropriate governance, and prospective demonstration that its use improves clinically meaningful outcomes [60,108]. At present, these approaches should therefore be regarded as research tools rather than established components of later-line decision-making.

Future development of adaptive precision oncology should consequently remain centered on clinically validated molecular information, rigorous prospective evidence, and methodologically robust RWE, with computational tools evaluated according to the same standards of clinical utility applied to other decision-support strategies.

## 6. Limitations

Several limitations should be considered when interpreting the evidence discussed in this narrative review. First, real-world evidence is inherently heterogeneous. Observational cohorts, registries, electronic health records, claims databases, and other real-world data sources differ in patient selection, data completeness, endpoint definitions, follow-up, and ascertainment of treatment exposure and outcomes. Missing data and inconsistent documentation may further affect comparability across studies [17,18,22]. Although methods such as multivariable adjustment, propensity-score approaches, inverse probability weighting, sensitivity analyses, and target-trial emulation can reduce important sources of bias, they cannot fully eliminate residual confounding or reproduce randomization [33,34,35,36,37].

These limitations are particularly relevant to comparative effectiveness and therapeutic sequencing. Patients who receive multiple later-line treatments are selected by survival, performance status, organ function, access to care, and previous treatment tolerance. Treatment attrition, immortal-time bias, time-dependent confounding, and confounding by indication may therefore influence apparent differences between therapeutic sequences. Observational associations should consequently not be interpreted as evidence of sequence superiority unless supported by appropriately designed prospective studies.

Selection effects are also important in precision oncology. Patients undergoing comprehensive molecular profiling, serial ctDNA testing, or treatment at specialized centers may have better performance status, longer survival, or greater access to subsequent therapies than the broader mCRC population. Favorable outcomes in biomarker-selected real-world cohorts may therefore reflect both treatment activity and characteristics of the selected population.

Longitudinal ctDNA assessment has additional limitations. Tumor DNA shedding varies according to disease burden and metastatic site, while assay sensitivity, genomic coverage, sampling intervals, and reporting thresholds differ across platforms [41,60]. A negative ctDNA result does not necessarily exclude resistant subclones. Accordingly, applications supported by prospective evidence, such as molecular selection for anti-EGFR rechallenge, should be distinguished from broader adaptive ctDNA-guided treatment strategies that remain investigational.

Finally, this is a narrative rather than a systematic review. Although the literature was extensively reviewed and greater interpretative weight was given to methodologically robust studies, no formal systematic-review protocol, quantitative evidence synthesis, or structured risk-of-bias scoring was applied. Study selection may therefore be susceptible to publication and selection bias. The breadth of the review, spanning multiple therapies and molecular subgroups, also limits the depth with which each individual strategy can be addressed.

## 7. Conclusions

The therapeutic landscape of refractory mCRC has evolved from a limited salvage setting to a more complex continuum of care that includes molecularly unselected later-line therapies, biomarker-directed treatments, immunotherapy for selected molecular subgroups, and ctDNA-informed anti-EGFR rechallenge [2]. As a result, treatment decisions increasingly require integration of previous exposure, residual fitness, cumulative toxicity, disease characteristics, molecular profile, and the feasibility of subsequent therapy.

Randomized trials remain the foundation for establishing treatment efficacy and safety. RWE provides complementary information by extending these findings to more heterogeneous populations and by characterizing treatment delivery, dose modification, toxicity, biomarker implementation, and patterns of care that may be difficult to evaluate fully in prospective trials. Its contribution is particularly relevant to external validity and routine-care implementation, whereas comparative effectiveness and sequencing analyses require greater caution because of selection bias, treatment attrition, time-dependent confounding, and residual confounding.

Precision oncology in later-line mCRC also requires a distinction between established and emerging applications of molecular information. Biomarker-directed treatments supported by prospective evidence and ctDNA-guided selection for anti-EGFR rechallenge can inform current clinical practice, whereas broader longitudinal ctDNA-guided treatment adaptation and computational decision-support strategies remain investigational.

The most informative approach is therefore not to view randomized evidence and RWE as competing sources, but to integrate them according to the clinical question being addressed. Rigorous RWE can help bridge the gap between trial efficacy and routine-care effectiveness and identify areas requiring prospective validation. As later-line treatment becomes increasingly individualized, this complementary evidence framework may support more informed therapeutic sequencing while preserving appropriate caution when observational associations cannot establish causality.

## Figures and Tables

**Table 1:** Practical molecular framework for precision oncology in later-line metastatic colorectal cancer.

Molecular Domain	Clinical Relevance in Third-Line+ mCRC	Role in Treatment Selection and Longitudinal Management	Clinical Evidence
**Core molecular profiling: RAS, *BRAF V600E*, MSI/MMR, HER2**	Defines major molecular subgroups with established therapeutic implications	Identifies eligibility for anti-EGFR strategies, *BRAF*-directed therapy, immune checkpoint inhibition and HER2-targeted treatment	[2,3]
**Broad NGS for additional actionable alterations**	Detects less frequent but therapeutically relevant alterations, including *KRAS G12C*, NTRK and *RET* fusions, and *POLE*/*POLD1* proofreading-domain mutations	Expands access to matched or tumour-agnostic therapies and identifies selected hypermutated phenotypes potentially relevant to immunotherapy	[2,4]
**ctDNA for molecular reassessment**	Provides a minimally invasive approach to updated molecular characterization when tissue is unavailable or rapid reassessment is clinically relevant	May identify evolving resistance mechanisms and refine eligibility for selected biomarker-driven strategies	[2]
**ctDNA-guided anti-EGFR rechallenge**	Assesses persistence or clearance of acquired resistance alterations after previous anti-EGFR exposure	Supports selection of patients for anti-EGFR rechallenge when resistant *RAS*/*BRAF*/EGFR-ECD clones are no longer detectable	[2,44,45,46,47,48]
**Longitudinal molecular profiling**	Recognizes that tumour biology evolves under treatment pressure	Integrates baseline and post-progression molecular information into adaptive sequencing and clinical-trial selection	[2,4]
**Molecular Tumor Board integration**	Provides multidisciplinary interpretation of complex or uncommon molecular findings	Links molecular actionability with prior treatment exposure, patient fitness, drug accessibility and trial opportunities	[2,4]

Abbreviations: ctDNA, circulating tumor DNA; EGFR, epidermal growth factor receptor; EGFR-ECD, epidermal growth factor receptor extracellular domain; HER2, human epidermal growth factor receptor 2; mCRC, metastatic colorectal cancer; MMR, mismatch repair; MSI, microsatellite instability; MSI, microsatellite instability; NGS, next-generation sequencing.

**Table 2:** Clinical and molecular determinants of later-line treatment sequencing in mCRC.

Sequencing Determinant	Clinical Assessment	Potential Impact on Treatment Sequencing	Clinical Evidence
**Actionable molecular profile**	MSI-H/dMMR, *BRAF V600E*, HER2, *KRAS G12C*, NTRK/*RET* and other actionable alterations	Prioritizes matched targeted therapy or immunotherapy when clinically appropriate and available; molecular status should be integrated with previous exposure and treatment goals	[2,3,4]
**Performance status, comorbidity and organ function**	ECOG PS, frailty, comorbidity burden, hepatic/renal function and marrow reserve	Favors strategies with a tolerability profile compatible with individual fitness and may determine whether the patient remains eligible for subsequent lines	[2,3]
**Attrition risk**	Clinical deterioration, rapidly declining PS, nutritional status and likelihood of reaching another treatment line	Supports earlier use of therapies with the greatest expected individual benefit rather than assuming that all active options can be delivered sequentially	[2]
**Disease burden and kinetics**	Tumour burden, pace of progression, symptoms and threatened organ function	A greater need for rapid tumour control may favor therapies with a higher probability of objective response or an immediately actionable biomarker-directed strategy	[2]
**Previous treatment exposure and benefit**	Prior chemotherapy, anti-VEGF/anti-EGFR therapy, targeted agents, duration of previous disease control and resistance pattern	Avoids ineffective repetition, informs rechallenge opportunities and helps construct biologically rational subsequent sequences	[2,3]
**Residual and cumulative toxicity**	Neuropathy, myelosuppression, fatigue, gastrointestinal toxicity, HFSR, hypertension and other persistent adverse events	Allows selection of treatments with non-overlapping or more manageable toxicity profiles and helps preserve the therapeutic trajectory	[2,3]
**Dynamic molecular reassessment**	Tissue re-biopsy and/or ctDNA when clinically informative	May identify molecular evolution, acquired resistance and renewed eligibility for selected strategies, particularly anti-EGFR rechallenge	[2,44,45,46,47,48]
**Treatment delivery and real-world feasibility**	Dose modifications, treatment discontinuation, adherence, testing turnaround time, access and logistical burden	Determines whether an evidence-based treatment can actually be delivered and sustained in routine practice and influences access to subsequent therapies	[2,22]
**Patient goals and preferences**	Expected benefit, toxicity acceptance, oral versus intravenous treatment, QoL priorities and treatment burden	Supports shared decision-making and aligns the therapeutic sequence with patient-centered goals	[2,3]

Abbreviations: ctDNA, circulating tumor DNA; ECOG PS, Eastern Cooperative Oncology Group performance status; EGFR, epidermal growth factor receptor; HER2, human epidermal growth factor receptor 2; HFSR, hand–foot skin reaction; mCRC, metastatic colorectal cancer; MSI-H/dMMR, microsatellite instability-high/mismatch repair deficient; QoL, quality of life; VEGF, vascular endothelial growth factor.

**Table 3:** Principal domains in which real-world evidence complements randomized evidence in later-line mCRC.

RWE Domain	Clinical Question Addressed in Routine Practice	Added Value Beyond Pivotal RCTs	Representative Application in Later-Line mCRC	Clinical Evidence
**External validity**	Do efficacy and safety observed in trials translate to patients who are older, frail, comorbid, or have poorer performance status?	Extends evidence to populations underrepresented or excluded from registration trials and characterizes heterogeneity of treatment effect in routine care	Outcomes and tolerability of later-line therapies in older, vulnerable and less-selected populations	[20,21,54,56]
**Treatment delivery**	How are therapies actually initiated, modified and maintained outside protocol-driven settings?	Captures starting dose, dose escalation/reduction, treatment duration, adherence, discontinuation and attrition to subsequent lines	Regorafenib dose optimization; FTD/TPI treatment delays/reductions and hematologic toxicity management	[20,21,30,49]
**Comparative effectiveness and sequencing**	Does treatment order influence cumulative benefit and access to subsequent therapies?	Enables comparison of real-world treatment trajectories when direct randomized sequence trials are unavailable or impractical, while requiring appropriate control of confounding	Regorafenib → FTD/TPI versus FTD/TPI → regorafenib and other later-line sequencing patterns	[30,55,57]
**Safety in routine practice**	How do toxicity and its management affect exposure, discontinuation and subsequent treatment eligibility?	Characterizes toxicity management in heterogeneous populations and links adverse events to actual treatment delivery	HFSR and dose modification with regorafenib; neutropenia, dose delays and treatment exposure with FTD/TPI	[20,21,58,59]
**Biomarker implementation**	Are molecular tests obtained at the right time, and do their results actually translate into treatment access?	Evaluates testing uptake, turnaround time, assay feasibility, tissue limitations and integration of ctDNA into clinical pathways	Molecular selection for targeted therapies and ctDNA-guided anti-EGFR rechallenge	[2,4,44,45,46,47,48]
**Learning loops and adaptive care**	Can longitudinal clinical and molecular data refine subsequent decisions as disease evolves?	Connects treatment decisions, outcomes and molecular evolution to iterative pathway refinement through registries and Molecular Tumor Boards	Longitudinal ctDNA, resistance monitoring, post-progression reassessment and adaptive sequencing	[2,60]

Abbreviations: ctDNA, circulating tumor DNA; FTD/TPI, trifluridine/tipiracil; HFSR, hand–foot skin reaction; mCRC, metastatic colorectal cancer; RCT, randomized controlled trial; RWE, real-world evidence.

**Table 4:** Key regorafenib evidence shaping real-world implementation and treatment sequencing in refractory mCRC.

Evidence/Setting	Main Contribution	Implications for Real-World Practice	Clinical Evidence
**CORRECT–phase III randomized trial**	Established the survival benefit of regorafenib versus placebo in previously treated mCRC	Established regorafenib as an active later-line option and provided the reference efficacy and safety framework for subsequent RWE	[7]
**CONCUR–phase III randomized trial**	Confirmed the efficacy of regorafenib in an Asian population with refractory mCRC	Supported the generalizability of regorafenib activity across different populations and treatment histories	[8]
**CONSIGN–phase IIIb, single-arm study**	Characterized safety and treatment delivery in a large population treated after standard therapy	Reinforced the importance of early toxicity recognition, monitoring, and dose modification	[21]
**CORRELATE–prospective observational study**	Evaluated effectiveness, safety, starting dose, and dose modifications under routine-practice conditions	Documented heterogeneity in real-world dosing and the feasibility of individualized treatment delivery	[20]
**ReDOS–randomized phase II dose-optimization study**	Compared dose escalation from 80 mg/day with standard 160-mg initiation	Provided prospective evidence supporting dose escalation as an alternative strategy to improve early treatment tolerability and continuation	[28]
**Additional evidence on dose optimization**	Evaluated alternative induction strategies and adoption of flexible dosing in clinical practice	Supports tolerability-oriented dose individualization while preserving treatment exposure	[63,64]
**Real-world sequencing studies**	Evaluated treatment trajectories involving regorafenib and FTD/TPI, including alternative treatment orders	Provides clinically relevant sequencing information, while observational comparisons remain susceptible to selection, attrition, and time-dependent biases	[30,55,57,67]

Abbreviations: FTD/TPI, trifluridine/tipiracil; mCRC, metastatic colorectal cancer; RWE, real-world evidence.

**Table 5:** Key evidence shaping FTD/TPI-based later-line treatment in refractory mCRC.

Evidence/Setting	Main Contribution	Implications for Clinical Practice	Clinical Evidence
**RECOURSE–phase III randomized trial**	Established the efficacy and safety of FTD/TPI monotherapy in refractory mCRC	Established FTD/TPI as an evidence-based later-line treatment	[9]
**Real-world FTD/TPI experience**	Extended evidence on effectiveness, tolerability, treatment delivery, and dose modification to less-selected populations	Helps characterize the feasibility of FTD/TPI in routine practice, including older and clinically heterogeneous patients	[38,58]
**Treatment-emergent neutropenia**	Linked greater FTD/TPI exposure and early neutropenia with improved clinical outcomes	Supports CIN as a potential pharmacodynamic marker of exposure, but not as a validated predictive biomarker	[59]
**SUNLIGHT–phase III randomized trial**	FTD/TPI plus bevacizumab improved OS and PFS versus FTD/TPI alone	Established FTD/TPI plus bevacizumab as a major later-line treatment option	[71]
**Post-SUNLIGHT RWE–TWILIGHT and multicenter cohorts**	Evaluated FTD/TPI plus bevacizumab in vulnerable or routine-care populations	Supports feasibility in less-selected populations and informs comparative treatment delivery while remaining non-randomized	[54,72]

Abbreviations: CIN, chemotherapy-induced neutropenia; FTD/TPI, trifluridine/tipiracil; mCRC, metastatic colorectal cancer; OS, overall survival; PFS, progression-free survival; RWE, real-world evidence.

**Table 6:** Key evidence supporting ctDNA-guided anti-EGFR rechallenge in refractory mCRC.

Evidence/Study	Main Contribution	Clinical Implication	Evidence
**Parseghian et al.–clonal decay**	Demonstrated decay of acquired RAS/EGFR-resistant clones after anti-EGFR withdrawal	Provided the biological rationale for rechallenge after an EGFR-free interval	[44]
**CRICKET–phase II**	Showed that absence of detectable RAS resistance alterations in baseline ctDNA was associated with improved outcome during cetuximab–irinotecan rechallenge	Supported molecular selection beyond clinical criteria alone	[45]
**CHRONOS–phase II**	Prospectively selected patients according to absence of RAS/*BRAF*/EGFR-ECD resistance alterations in ctDNA	Established prospective ctDNA-guided selection for chemotherapy-free anti-EGFR rechallenge	[14]
**PARERE–randomized phase II**	Evaluated panitumumab and regorafenib sequencing in ctDNA RAS/*BRAF* wild-type patients	Extended rechallenge evidence to a randomized later-line sequencing strategy	[46]
**Pooled Italian rechallenge analysis**	Evaluated anti-EGFR rechallenge in molecularly selected ctDNA RAS/*BRAF* wild-type patients	Supported activity in molecularly selected patients and the potential value of broader negative selection	[47]
**RASINTRO–prospective multicenter study**	Evaluated baseline ctDNA status and early ctDNA changes during rechallenge	Supports further investigation of dynamic molecular monitoring beyond baseline selection	[48]

Abbreviations: ctDNA, circulating tumor DNA; EGFR, epidermal growth factor receptor; EGFR-ECD, epidermal growth factor receptor extracellular domain; mCRC, metastatic colorectal cancer.

**Table 7:** Biomarker-driven targeted therapies in refractory mCRC: evidence and real-world implementation.

Molecular Subgroup	Established/Supported Strategy	Evidence Status	Main Real-World Question	Clinical Evidence
**BRAF V600E**	Encorafenib + anti-EGFR therapy	Randomized phase III evidence	External validity, treatment delivery, sequencing, and post-progression pathways	[2,97,99]
***KRAS G12C***	Sotorasib + panitumumab; adagrasib + cetuximab	Randomized phase III and prospective clinical evidence	Molecular testing, access, toxicity management, resistance, and subsequent treatment	[103,105]
**NTRK fusion**	TRK-directed therapy	Tumor-agnostic prospective/basket evidence	Detection by broad NGS, access, durability, and treatment after resistance	[2,107]
***RET*** **fusion**	Selpercatinib	Tumor-agnostic evidence/guideline-supported option	Molecular detection, access, and long-term effectiveness in an ultra-rare subgroup	[2]
**Rare actionable alterations**	Molecularly matched therapy according to the alteration	Evidence varies by target; often basket-trial based	Registries can complement prospective evidence by characterizing implementation, toxicity, and resistance	[2,22,104]

Abbreviations: BRAF, B-Raf proto-oncogene, serine/threonine kinase; EGFR, epidermal growth factor receptor; KRAS, Kirsten rat sarcoma viral oncogene homolog; NTRK, neurotrophic tyrosine receptor kinase; TRK, tropomyosin receptor kinase; *RET*, rearranged during transfection; NGS, next-generation sequencing.

## Data Availability

No new data were created or analyzed in this study. Data sharing is not applicable to this article.

## Abbreviations

The following abbreviations are used in this manuscript:

3L+	third-line and later
ADC	antibody–drug conjugate
AE	adverse event
AI	artificial intelligence
BRAF	B-Raf proto-oncogene
CIN	chemotherapy-induced neutropenia
CNS	central nervous system
CRC	colorectal cancer
ctDNA	circulating tumor DNA
DCR	disease control rate
dMMR	mismatch repair deficient
ECD	extracellular domain
ECOG PS	Eastern Cooperative Oncology Group performance status
EGFR	epidermal growth factor receptor
EGFR-ECD	epidermal growth factor receptor extracellular domain
EGFRi	epidermal growth factor receptor inhibitor
EHR	electronic health record
ESMO	European Society for Medical Oncology
ESMO-GROW	ESMO Guidance for Reporting Oncology real-World evidence
FTD/TPI	trifluridine/tipiracil
HER2	human epidermal growth factor receptor 2
HFSR	hand–foot skin reaction
HRQoL	health-related quality of life
ICI	immune checkpoint inhibitor
IHC	immunohistochemistry
ILD	interstitial lung disease
IO	immuno-oncology
IPW	inverse probability weighting
KRAS	Kirsten rat sarcoma viral oncogene homolog
mAb	monoclonal antibody
mCRC	metastatic colorectal cancer
MMR	mismatch repair
MSI	microsatellite instability
MSI-H	microsatellite instability-high
MSS	microsatellite stable
MTB	Molecular Tumor Board
NCCN	National Comprehensive Cancer Network
NGS	next-generation sequencing
NRAS	neuroblastoma RAS viral oncogene homolog
NTRK	neurotrophic tyrosine receptor kinase
ORR	objective response rate
OS	overall survival
PD-1	programmed cell death protein 1
PFS	progression-free survival
pMMR	mismatch repair proficient
POLE/POLD1	DNA polymerase epsilon/delta 1
PROs	patient-reported outcomes
PS	performance status
PSM	propensity score matching
QoL	quality of life
RAS	rat sarcoma virus oncogene homolog
RCT	randomized controlled trial
RECIST	Response Evaluation Criteria in Solid Tumors
RET	rearranged during transfection
RWD	real-world data
RWE	real-world evidence
rwTTD	real-world time to treatment discontinuation
SOC	standard of care
T-DXd	trastuzumab deruxtecan
TKI	tyrosine kinase inhibitor
TRK	tropomyosin receptor kinase
TTD	time to treatment discontinuation
VEGF	vascular endothelial growth factor
VEGFR	vascular endothelial growth factor receptor
